# Thermal ablation vs surgical resection for ultrasound-detected T2N0M0 papillary thyroid carcinoma

**DOI:** 10.1093/oncolo/oyaf170

**Published:** 2025-06-30

**Authors:** Yu-tong Liu, Ying Wei, Zhen-Long Zhao, Jie Wu, Shi-Liang Cao, Na Yu, Yan Li, Li-Li Peng, Ming-an Yu

**Affiliations:** China-Japan Friendship Hospital (Institute of Clinical Medical Sciences), Chinese Academy of Medical Sciences & Peking Union Medical College, Beijing 100029, China; Department of Interventional Medicine, China-Japan Friendship Hospital, Beijing 100029, China; Department of Interventional Medicine, China-Japan Friendship Hospital, Beijing 100029, China; Department of Interventional Medicine, China-Japan Friendship Hospital, Beijing 100029, China; Department of Interventional Medicine, China-Japan Friendship Hospital, Beijing 100029, China; Department of Interventional Medicine, China-Japan Friendship Hospital, Beijing 100029, China; Department of Interventional Medicine, China-Japan Friendship Hospital, Beijing 100029, China; Department of Interventional Medicine, China-Japan Friendship Hospital, Beijing 100029, China; Department of Interventional Medicine, China-Japan Friendship Hospital, Beijing 100029, China

**Keywords:** papillary thyroid carcinoma, thermal ablation, surgical resection, disease progression, comparative study

## Abstract

**Background:**

Thermal ablation (TA) has demonstrated promising efficacy and safety in treating papillary thyroid carcinoma (PTC). However, comparative analyses between TA and surgical resection (SR) for treating T2N0M0 PTC remain scarce.

**Purpose:**

To compare the efficacy and safety of TA and SR in treating preoperatively US-detected T2N0M0 PTC.

**Materials and Methods:**

In this retrospective study, 252 patients with preoperative ultrasound (US)-detected T2N0M0 PTC treated by TA or SR between January   2016 and May 2024 were included. Comparative study based on propensity score matching (PSM) between the TA and SR groups was conducted.

**Results:**

After PSM, 63 patients (median age: 40 years [IQR 34-53], 49 females) in the TA group and 126 patients (median age: 41 years [IQR 32-52.5], 102 females) in the SR group were followed for a median of 35 months (IQR 18-62) and 46 months (IQR 29-60), respectively (*P* = .093). There was no evidence of differences in disease progression between the TA and SR groups (11.1% vs 7.9%; *P* = .59) or progression-free survival rates between the TA and SR groups (86.4% vs 89.6%, *P* = .37). Compared with SR, TA resulted in less blood loss, a shorter incision length, and shorter procedure and hospitalization times (all, *P* < .001). Although there was a relatively higher incidence of hoarseness in the TA group, no significant difference was observed between the TA and SR groups (12.7% vs 8.7%, *P* = .444) or between the TA and lobectomy (ie, hemithyroidectomy and subtotal thyroidectomy) groups (12.7% vs 5.9%, *P* = .340).

**Conclusions:**

There were no significant differences in disease progression and hoarseness between TA and SR for US-detected T2N0M0 PTC. Thermal ablation is an effective and safe treatment option for US-detected T2N0M0 PTC.

**Implications for Practice**

Thermal ablation (TA) demonstrates comparable efficacy to surgical resection (SR) for treating US-detected T2N0M0 papillary thyroid carcinoma (PTC), making it a valid alternative for appropriate patients. Thermal ablation is associated with reduced blood loss, smaller incisions, and shorter procedure and hospitalization times compared with SR, which can lead to faster recovery and lower perioperative morbidity. With no significant differences in major complications or progression-free survival between TA and SR, clinicians may consider TA as a safe and efficacy option for patients with T2N0M0 PTC. These findings demonstrated the incorporation of TA into clinical practice, particularly for patients who are either high-risk for surgery or prefer minimally invasive procedures.

## Introduction

Thyroid cancer is the 10th most common cancer worldwide, with papillary thyroid carcinoma (PTC) accounting for 80%-90% of all differentiated thyroid carcinomas.^1^ The American Thyroid Association Management Guidelines recommend total thyroidectomy or lobectomy as the first-line treatment for patients with PTC.^2^ However, the potential drawbacks of thyroidectomy, such as hypothyroidism and the possible lifelong need for levothyroxine therapy, can significantly impact a patient’s quality of life.^3-5^ These drawbacks of thyroidectomy for PTC cannot be ignored. Additionally, considering the indolent nature of PTC and the high 10-year survival rate (exceeding 90%),^1,6,7^ the management and treatment options for PTC should be re-evaluated.

Currently, recent studies suggest that ultrasound (US)-guided thermal ablation (TA), mostly including microwave ablation (MWA) and radiofrequency ablation (RFA), is a minimally invasive, safe, and effective method for treating PTC.^8-10^ Several studies comparing TA with surgical resection (SR) for PTC in the T1a or T1 stage suggest that TA is as effective as SR.^9-12^ This makes TA a viable alternative for patients who are either unsuitable candidates for surgery or opt against it, offering a promising treatment option.^8-12^ According to thyroid cancer stage, T2N0M0 PTC is defined as a tumor size of 2-4 cm without lymph node metastasis (LNM) or distant metastasis (DM).^1^ To date, there have been few studies on TA for T2N0M0 PTC^13,14^; one previous study involving 34 patients with T2N0M0 PTC demonstrated that TA is both effective and safe for treating these patients.^14^ However, there are still no definitive comparative studies assessing the therapeutic efficacy and safety of TA vs SR for the treatment of T2N0M0 PTC.

Therefore, the present retrospective study aimed to compare the efficacy and safety of TA vs SR in treating T2N0M0 PTC in terms of technical effectiveness, disease progression, and complications.

## Methods

**Figure 1.**
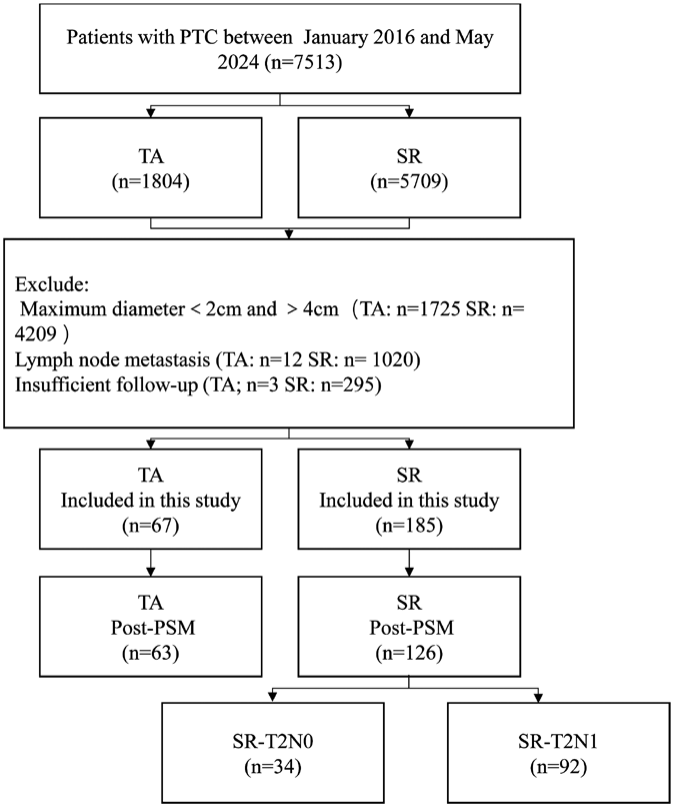
Patient flowchart. Abbreviations: PSM, propensity score matching; PTC, papillary thyroid carcinoma; SR, surgical resection; TA, thermal ablation.

This retrospective study was approved at our hospital, with the protocol approved by the Human Ethics Review Committees. Data were collected from patients who underwent TA or SR for PTC between January 2016 and May 2024. The requirement for written informed consent for publication was waived due to the retrospective nature of the study. The inclusion criteria were as follows: (1) PTC confirmed by fine needle aspiration (FNA) or core-needle biopsy (CNB), with a maximum diameter (MD) >2 cm and ≤4 cm; (2) solitary and unilateral T2N0M0 PTC with no contralateral PTC detected on US; (3) no evidence of extrathyroidal invasion, LNM or DM on preoperative US or chest CT, confirming preoperative T2N0M0; (4) for patients in the TA group, ineligibility or refusal of surgery (the criterion for surgical ineligibility was the inability to tolerate general anesthesia or tracheal intubation); and (5) no prior thyroid surgery. The exclusion criteria were as follows:(1) severe coagulation disorder (platelet count < 60 × 109/L or prothrombin activity <60%) or organ failure; (2) insufficient clinical data; or (3) follow-up of less than 12 months (Figure 1).

### Preoperative assessment

Before treatment, the US was used to assess the patients. Details of the preoperative assessment are presented in Supplementary Material 1. In the TA and SR groups, the suspicious nodules underwent biopsy.

### Treatment

**Figure 2.**
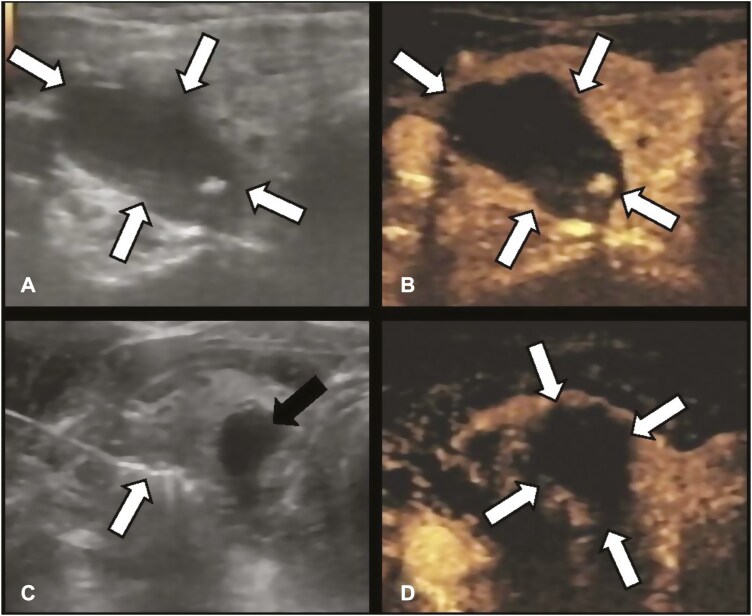
Ablation procedure was performed on a 50-year-old female with T2N0M0 PTC (size: 2.8 × 2 × 1.5 cm). (A) Routine US shows a hypoechoic T2N0M0 PTC nodule (white arrows) in the left lobe. (B) Preablation contrast-enhanced US image shows hypo-enhancement in the arterial phase (white arrows). (C) Isolating fluid (black arrow) was performed to protect the adjacent critical structure (white arrow) during ablation. (D) Postablation contrast-enhanced US image shows no enhancement in the original tumor zone (white arrows).

Thermal ablation was performed using a US-guided ablation technique.^12,15,16^ For further details on the TA and SR procedures, see Supplementary Material 2. Representative US images of the T2N0M0 PTC ablation procedure are depicted in Figure 2.

### Postoperative assessment and follow-up

After TA or SR, demographics, tumor characteristics, procedure time, incision length, estimated blood loss, and hospitalization time were recorded. Follow-up was performed every three months during the first year and every 6 months thereafter. US examination and thyroid function tests (normal reference ranges: FT3, 2.0-4.4 pg/mL; FT4, 0.93-1.7 ng/dL; TSH, 0.27-4.2 µIU/mL) were performed at every follow-up session. CT of the neck and chest was performed annually to monitor for LN and DM. For suspected tumor recurrence and LNM, FNA or CNB was performed for confirmation. Complications, including major complications (ie, transient and permanent hoarseness), minor complications (ie, hematoma), and side effects (ie, fever and transient hypoparathyroidism) were recorded. Permanent recurrent laryngeal nerve injury was defined as an injury at least 6 months after treatment.^12^ Postoperative hypoparathyroidism was defined as biochemical hypoparathyroidism (PTH < 12 pg/mL) accompanied by symptoms and/or signs of hypocalcemia, with recovery occurring within 6 months considered to be transient; otherwise, the condition was considered to be permanent.^17^ The volume reduction ratio (VRR) was calculated using the following formula: VRR = ([initial volume − final volume]/initial volume) × 100%.

### Clinical outcomes and definitions

The primary outcomes were the disease progression, technical success, and complications. The secondary outcomes included changes in tumor size and volume after TA, as well as comparisons between TA and SR in terms of procedure time, estimated blood loss, incision length, hospitalization time, and the need for thyroid hormone replacement therapy (TRT).

Disease progression was classified into 5 categories: (1) local recurrence, (2) new tumor, (3) LNM, (4) DM, and (5) death associated with PTC (see Supplementary Material 3). Technical success was defined as a complete absence of enhancement on the target tumor in CEUS at the end of each procedure.^18^

### Statistical methods

Descriptive statistics for continuous variables were presented as medians and interquartile ranges (IQR). Categorical variables were presented as frequency and percentage. Propensity score matching (PSM) with a 1:2 ratio was performed to balance baseline characteristics (sex, age, and MD of the PTC) in the TA and SR groups before comparison. The caliper for PSM was 0.2. The Wilcoxon rank-sum test or *t*-test was used for continuous variables and the *χ*^2^ test or Fisher exact test was used for categorical variables. The Kaplan–Meier method was used to plot survival curves for disease progression rates, and differences between the TA and SR groups were assessed using the log-rank test. All statistical analyses and diagrams were conducted using the R software (version 4.3.1). *P* < .05 was indicative of a significant difference.

## Results

### Demographic characteristics

Data from 7513 patients with PTC who underwent TA (1804 patients) or SR (5709 patients) between July 2016 and May 2024 were reviewed. After the exclusion of 7260 patients who did not meet the inclusion criteria, 252 patients were included. Before PSM, 67 patients (median age, 38 years [IQR: 32-51]; 52 females) underwent TA, and 185 patients (median age, 41 years [IQR: 32-52.5]; 125 females) underwent SR. After PSM with a 1:2 ratio, 63 patients were included in the TA group, and 126 patients were included in the SR group (Figure 1). The median follow-up durations for the TA group and SR group were 35 months (IQR, 18-62 months) and 46 months (IQR, 29-60 months), respectively (*P* = .093). All 126 patients in the SR group underwent CLND. According to postoperative pathology, 27% (34/126) SR patients were placed in the SR-T2N0 subgroup, and 73% (92/126) in the SR-T2N1 subgroup (Figure 1). All 63 patients in the TA group after PSM were used for comparisons with the SR group, as well as the SR-T2N0 and SR-T2N1 subgroups. The demographic characteristics are summarized in Table 1 and Table S1 (see Supplementary Material 4).

**Table 1.** Baseline patient characteristics before and after PSM.

	Before PSM			After PSM		
Variable	TA (*n* = 67)	SR (*n* = 185)	*P*-value	TA (*n* = 63)	SR (*n* = 126)	*P*-value
Age (years)	38 (32,51)	41 (32,52.5)	.820	40 (34,53)	41 (32,52.5)	.903
Sex			.160			.608
Male	15 (22.4%)	60 (32.4%)		14 (22.2%)	24 (19%)	
Female	52 (77.6%)	125 (67.6%)		49 (77.8%)	102 (81%)	
MD (cm)	2.5 (2.3,2.9)	2.5 (2.5,3)	.082	2.5 (2.3,3)	2.5 (2.3,2.9)	.595

Data are medians with interquartile ranges in parentheses for continuous variables and are numbers of patients with percentages in parentheses for categorical variables. Abbreviations: MD, maximum diameter; PSM, propensity score matching; SR, surgical resection; TA, thermal ablation.

### Treatment variables

In the TA group, all 63 cases underwent ablation in a single session, with complete absence of enhancement on CEUS observed in all target tumors. In the SR group, 75 cases (59.5%) underwent total thyroidectomy, 39 cases (31%) underwent hemithyroidectomy, and 12 cases (9.5%) underwent subtotal thyroidectomy. The technical success rate was 100% in the TA and SR groups.

Compared with the SR group, the TA group had shorter procedure time (median 34 min [IQR, 33-35] vs 92.5 min [IQR, 60-120]), shorter hospitalization time (median 3 days [IQR, 3-3] vs 5 days [IQR, 4-6]), less estimated blood loss (median 4 mL [IQR, 2-6] vs 10 mL [IQR, 10-20]) and shorter incision length (median 0.2 cm [IQR, 0.1-0.2] vs 8 cm [IQR, 7–8]) (all, *P* < .001). At the 6-month postoperative follow-up, FT3 (TA vs SR: median 3.14 pg/mL [IQR, 2.91-3.54] vs 3.05 pg/mL [IQR, 2.82-3.30]; *P* = .16), FT4 (TA vs SR: median 1.34 ng/dL [IQR, 1.18-1.46] vs 1.52 ng/dL [IQR, 1.32-1.68]; *P* < .001), and TSH levels (TA vs SR: median 1.48 µIU/mL [IQR, 0.95-2.28] vs 1.09 µIU/mL [IQR, 0.27-2.07]; *P* = .042) remained within normal reference ranges in both groups, with TRT only being administered in the SR group. There was a significant difference in TRT between the 2 groups [TA vs SR = 0 (0/63) vs 92.9% (117/126); *P* < .001] (Table 2).

**Table 2.** Treatment variables in the TA and SR groups.

Variable	TA (*n* = 63)	SR (*n* = 126)	*P*-value
Procedure time (min)	34 (33,35)	92.5 (60,120)	<.001
hospitalization time (day)	3 (3,3)	5 (4,6)	<.001
Estimated blood loss (mL)	4 (2,6)	10 (10,20)	<.001
Incision length (cm)	0.2 (0.1,0.2)	8 (7,8)	<.001
Postoperative FT3 (pg/mL)	3.14 (2.91, 3.54)	3.05 (2.82, 3.30)	.160
Postoperative FT4 (ng/dL)	1.34 (1.18, 1.46)	1.52 (1.32, 1.68)	<.001
Postoperative TSH (uIU/mL)	1.48 (0.95, 2.28)	1.09 (0.27, 2.07)	.042
TRT	0 (0%)	117 (92.9%)	<.001

Data are medians with interquartile ranges in parentheses for continuous variables and are numbers of patients with percentages in parentheses for categorical variables. Abbreviations: FT3, free triiodothyronine; FT4, free thyroxine; MD, maximum diameter; PSM, propensity score matching; SR, surgical resection; TA, thermal ablation; TRT, thyroid hormone replacement therapy; TSH, thyroid-stimulating hormone.

### Tumor changes before and after TA

Because of expanded ablation, the mean VRR (−14%) after TA was negative within 1 month. There was no evidence of a difference in the MD and volume between before TA and 1 month after TA (median 2.5 cm [IQR, 2.3-2.9] vs 2.7 cm [IQR, 2.4-3.1], *P* = .283; median 3.7 mL [IQR, 2.8-6.4] vs 4.4 mL [IQR, 3.1-6.3], *P* = .987). There was a significant difference in the MD and volume between before and after TA during 3, 6, 9, 12, 24, and 36 months (all *P* < 0.001). The MD and volume of the ablation zone gradually decreased with each follow-up (Supplementary Material 5; Table S2).

### Complications

The incidence of major complications was 12.7% (8/63) in the TA group and 8.7% (11/126) in the SR group (*P* = .444). No significant difference was observed in transient hoarseness between the TA and SR groups [12.7% (8/63) vs 4.8% (6/126); *P* = .074]. Although the permanent hoarseness only occurred in the SR group, there was no evidence of a difference in the incidence of permanent hoarseness between the TA and SR groups [0% (0/63) vs 4% (5/126); *P* = .171]. In the TA group, all 8 patients with transient hoarseness spontaneously resolved within 6 months without any treatment. For minor complications and side effects analysis, the incidences of hematoma, fever, and transient hypoparathyroidism in the SR group were 4% (5/126), 2.4% (3/126), and 4% (5/126), respectively. There was no occurrence of patients with minor complications or side effects in the TA group. However, there was no evidence of a difference in the minor complications [0% (0/63) vs 4% (5/126); *P* = .171] or side effects [0% (0/63) vs 6.3% (8/126); *P* = .054] between the TA and SR groups (Table 3).

**Table 3.** Complications in the TA and SR groups.

Complication	TA (*n* = 63)	SR (*n* = 126)	*P*-value	Lobectomy (*n* = 51)	*P*-value
Major complications	8 (12.7%)	11 (8.7%)	.444	3 (5.9%)	.340
Transient hoarseness	8 (12.7%)	6 (4.8%)	.074	2 (3.9%)	.181
Permanent hoarseness	0	5 (4%)	.171	1 (2%)	.447
Minor complication	0	5(4%)	.171	3 (5.9%)	.087
Hematoma	0	5 (4%)	.171	3 (5.9%)	.087
Side effect	0	8 (6.3%)	.054	3 (5.9%)	.087
Fever >38 °C	0	3 (2.4%)	.552	3 (5.9%)	.087
Transient hypoparathyroidism	0	5 (4%)	.171	0	

Data are numbers of patients with percentages in parentheses for categorical variables. Abbreviations: SR, surgical resection; TA, thermal ablation.

A subgroup analysis comparing TA (*n* = 63) with lobectomy (ie, hemithyroidectomy and subtotal thyroidectomy) (*n* = 51) revealed no significant differences between the TA and lobectomy groups regarding major complications [12.7% (8/63) vs 5.9% (3/51); *P* = .34], minor complications [0% (0/63) vs 5.9% (3/51); *P* = .087], or side effects [0% (0/63) vs 5.9% (3/51); *P* = .087] (Table 3).

### Disease progression

The disease progression rate was 11.1% (7/63) in the TA group, which included local recurrence in 1 case (1.6%), new tumors in 3 cases (4.8%), and LNM in 3 cases (4.8%). The disease progression rate was 7.9% (10/126) in the SR group, which included new tumors in 4 cases (3.2%) and LNM in 9 cases (7.1%); among them, new tumors and LNM occurred in 3 cases simultaneously during postoperative follow-up. There was no evidence of a difference in disease progression between the TA and SR groups (11.1% vs 7.9%, *P* = .590). There was also no evidence of a difference in local recurrence, new tumors, and LNM between the TA and SR groups (1.6% vs 0%, *P* = .333; 4.8% vs 3.2%, *P* = .688; 4.8% vs 7.1%, *P* = .754, respectively). For SR subgroup analysis, the disease progression rate was 2.9% (1/34) in the SR-T2N0 subgroup, which included LNM in 1 case (2.9%). The disease progression rate was 9.8% (9/92) in the SR-T2N1 subgroup, which included new tumors in 4 cases (4.3%) and LNM in 8 cases (8.7%). There were no significant differences in the incidence of disease progression between the TA and SR-T2N0 groups [11.1% vs 2.9%; *P* = .254] or the TA and SR-T2N1 groups [11.1% vs 9.8%; *P* = 1.000]. There was also no significant difference in new tumors or LNM between the TA and SR-T2N0 groups [4.8% vs 4.3%, *P* = .310; 4.8% vs 2.9%; *P* = .754] or between the TA and SR-T2N1 [4.8% vs 4.3%, *P* = 1.000; 4.8% vs 8.7%, *P* = .527] (Table 4).

**Table 4.** Disease progression in the TA and SR groups.

Outcome	TA (*n* = 63)	SR (*n* = 126)	*P*-value	SR-T2N0 (*n* = 34)	*P*-value	SR-T2N1 (*n* = 92)	*P*-value
Disease progression	7 (11.1%)	10 (7.9%)	.590	1 (2.9%)	.254	9 (9.8%)	1.000
Local recurrence	1 (1.6%)	0	.333	0	1.000	0	.406
New tumors	3 (4.8%)	4 (3.2%)	.688	0	.310	4 (4.3%)	1.000
LNM	3 (4.8%)	9 (7.1%)	.754	1 (2.9%)	1.000	8 (8.7%)	.527

Data are numbers of patients with percentages in parentheses for categorical variables. Abbreviations: LNM, lymph node metastasis; SR, surgical resection; TA, thermal ablation.

**Figure 3.**
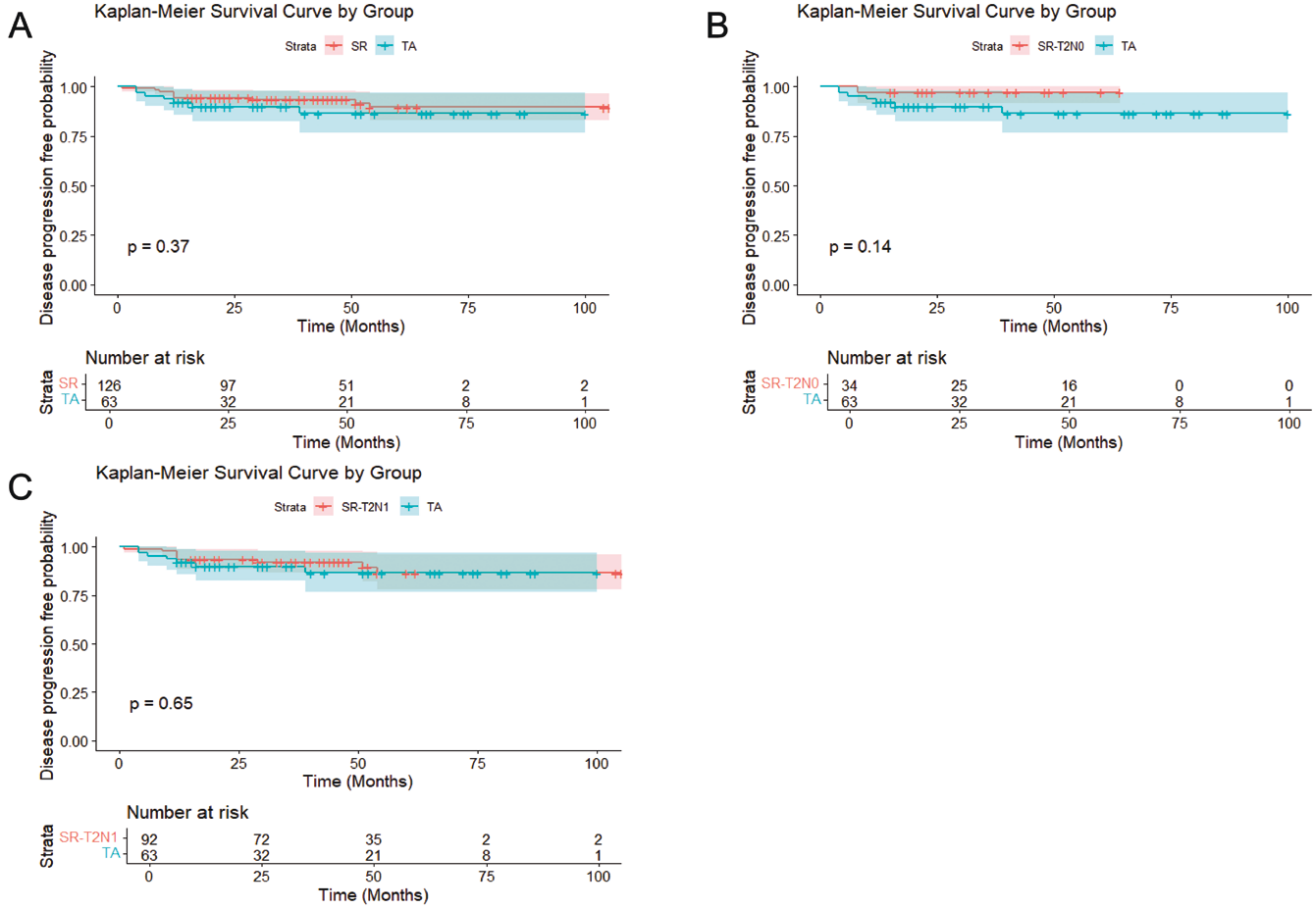
Kaplan–Meier survival curves based on PSM for overall disease progression in the TA and SR groups. (A) TA vs SR group. (B) TA vs SR-T2N0 subgroup. (C) TA vs SR-T2N1 subgroup.

According to the Kaplan–Meier survival analysis and log-rank test results, there was no evidence of a difference in the overall disease progression-free survival rates between the TA and SR groups (86.4% vs 89.6%, log-rank *P* = .37) or between the TA group and the SR-T2N0 (97.1% vs 86.4%, log-rank *P* = .14) or SR-T2N1 subgroup (86.4% vs 86.6%, log-rank *P* = .65). There was also no evidence of a difference in the 1-, 3-, or 5-year disease progression-free survival rates between the TA and SR groups (1-year: 93.4% vs 94.4%, *P* = .09; 3-year: 92.1% vs 89.6%, *P* = .812; 5-year: 86.4% vs 90%, *P* = .09) or between the TA group and the SR-T2N0 (1-year: 97.1% vs 97.1%, *P* = .277; 3-year: 92.1% vs 97.1%, *P* = .39; 5-year: 86.4% vs 90%, *P* = .06) or the SR-T2N1 subgroup (1-year: 92.1% vs 93.5%, *P* = .133; 3-year: 92.1% vs 86.5%, *P* = .93; 5-year: 86.4% vs 90%, *P* = .214) (Figure 3).

## Discussion

To date, although TA has been proven to be an effective and safe treatment for T1-stage PTC,^8-12^ evidence regarding the use of TA as an alternative to SR for the treatment of T2N0M0 PTC remains insufficient. Therefore, this study focused on comparing TA and SR for treating T2N0M0 PTC. In the present study, no significant difference in disease progression was observed between the TA and SR groups in the treatment of T2N0M0 PTC. However, the TA group exhibited less blood loss, shorter hospitalization times, shorter procedure times, and shorter incision lengths than the SR group.

In the present study, the technical success rate was 100% in the TA and SR groups. The disease progression rates as a primary outcome were 11.1% in the TA group and 7.9% in the SR group. Kaplan–Meier survival analysis showed no significant difference in the 1-year, 3-year, 5-year, or overall disease progression-free survival rates between the TA and SR groups, which is consistent with the previous studies demonstrating no significant difference in disease progression-free survival between TA and SR for T1-stage PTC.^9,18^ The above results suggest that TA is an effective treatment option for patients with T2N0M0 PTC.

Postoperative LNM was one of the major disease progressions. In the present study, the LNM rate in the TA group was 4.8%, which is higher than the rates of 0% to 2.2% reported in previous studies for T1-stage PTC.^18,25,26^ Notably, a tumor size greater than 2 cm has been identified as a significant risk factor for LNM in CN0 patients,^27^ which may contribute to the relatively high incidence of LNM after TA. However, the patients with LNM in the TA group underwent successful additional ablation for the LNM focus, and no further recurrence was observed during the follow-up period. In the SR group, after prophylactic CLND, the LNM rate remained as high as 7.9%. Based on the pathology results of prophylactic CLND, the SR group was divided into the SR-T2N0 and SR-T2N1 subgroups. No significant difference in postoperative LNM was observed between the TA and SR-T2N0 groups (4.8% vs 2.9%; *P* = 1.000) or between the TA and SR-T2N1 groups (4.8% vs 8.7%; *P* = .527). Although prophylactic CLND can remove parts of occult metastases, it does not seem to prevent regional recurrence,^28^ especially given the higher incidence of LNM in the SR-T2N1 group. Although there was no evidence of a difference in the LNM between the TA and SR groups, the LNM rate was lower in the TA group than in the SR group (4.8% vs 7.1%; *P* = .754). This finding suggests that undergoing TA without management of possible occult lymph node metastases has no negative impact on prognosis. However, in the present study, LNM was still observed in the SR group (despite the use of CLND). This finding suggests that CLND may not completely remove occult metastatic lymph nodes, thus underscoring the ongoing controversy regarding the necessity of routine prophylactic CLND in CN0 PTC patients.^29-31^ Therefore, decisions regarding the performance of CLND should be carefully individualized based on patient-specific factors.

In the present study, the new tumor rate in the TA group was 4.8%, which is within the previously reported range for MWA (1.8%-8.3%)^32,33^ and higher than that in the SR group of 3.2%. However, there was no significant difference in the new tumor between the TA and SR groups (*P* = .688). According to previous literature, SR reduces the likelihood of developing new tumors, especially with total thyroidectomy.^34^ However, the preoperative US does not detect micro-PTC lesions, which could contribute to the development of new tumors.^35^ Consequently, this limitation may explain why the TA and SR groups had the occurrence of new tumor during the follow-ups. Notably, in the TA group, local recurrence occurred in one patient. This patient’s tumor was relatively large (MD = 3.2 cm), which likely resulted in local recurrence due to the growth of residual tumor cells after a single ablation procedure. However, patients in the TA group who developed new tumors or local recurrences underwent additional ablation, and no further tumor progression was observed during the follow-ups. These results suggest that TA may still be used as a retreatment option for patients with new tumors or local recurrence.

The major complications observed in the present study included transient and permanent hoarseness. The transient hoarseness rate in the TA group was 12.7%, which is higher than the rates of 0% to 5.4% reported in previous studies for T1-stage PTC.^9,12,32^ Due to the larger tumor diameter in stage T2 compared to stage T1, the tumor is more likely to be closer to the recurrent laryngeal nerve, which increases the risk of healing injury to the nerve.^36^ In the SR group, the transient hoarseness rate was 4.8%, which is consistent with the results of previous studies that reported rates ranging from 1.6% to 5.3%.^19,23,37^ No significant difference in transient hoarseness was observed between the TA and SR groups (*P* = .074). There was also no significant difference in the incidence of transient hoarseness between the TA and lobectomy groups (12.7% vs 3.9%, *P* = .181). However, the patients with transient hoarseness in the TA group recovered within 6 months without any treatment, with no cases of permanent hoarseness being observed during the follow-up period. Although most of cases of transient hoarseness in the TA group were self-limiting, this relatively high incidence warrants clinical attention. In the SR group, the permanent hoarseness rate was 4%. This finding suggests that the use of the hydrodissection technique during ablation could effectively protect the recurrent laryngeal nerve, thus minimizing the risk of permanent damage.^12^ In the present study, no minor complications or side effects were observed in the TA group. Therefore, compared to SR, TA demonstrates better safety.

In the present study, during the follow-up, the volume and size of the nodules were significantly lower than those before ablation (all *P* < .01). The mean VRR at 12 months was 76.6%, which is consistent with previous studies reporting a VRR of 73.5% to 78.69% at 12 months.^21,24^ When the VRR exceeds 50%, it significantly alleviates the patient’s compression symptoms caused by thyroid tumors. Patients requiring lifelong thyroid hormone medication were observed only in the SR group, especially those who underwent total thyroidectomy. However, patients with PTC who undergo TA do not require lifelong medicines for hypothyroidism. At the 6-month postoperative follow-up, thyroid function (including FT3, FT4, and TSH measurements) remained within normal ranges for patients in both the TA (without TRT) and SR groups (with TRT). These findings suggest that TA effectively preserves thyroid function in patients with PTC.

A few limitations should be acknowledged. First, as a retrospective, single-center study, the results may be subject to selection bias and institutional practice-related limitations, despite the use of PSM. Second, pathologic subtype information for PTC was unavailable in the TA group, thereby limiting further stratified analyses. Third, the relatively small sample size and limited follow-up duration of this study may reduce the statistical power and restrict long-term outcome assessments. Future multicenter prospective studies with larger cohorts and longer follow-up periods are warranted to validate these findings.

In conclusion, TA has demonstrated safety and efficacy in treating T2N0M0 PTC, particularly offering advantages such as shorter procedure and hospitalization times, improved cosmetic outcomes, fewer complications, and the avoidance of lifelong thyroid hormone medication. However, the reported 12.7% rate of transient hoarseness after TA in T2N0M0 PTC should be carefully considered.

## Supplementary Material

oyaf170_suppl_Supplementary_Materials_1-5_Tables_S1-S2_Figures_S40-S47

## Data Availability

The datasets generated or analyzed during the study are available from the corresponding author on reasonable request.
