# Supplementary material for: Thermal ablation vs surgical resection for ultrasound-detected T2N0M0 papillary thyroid carcinoma
Source: Oncologist. 2025 Jun 30;30(6):oyaf170. doi: 10.1093/oncolo/oyaf170 (PMC12207881; doi:10.1093/oncolo/oyaf170)
Supplement: oyaf170_suppl_Supplementary_Materials_1-5_Tables_S1-S2_Figures_S40-S47 [file oyaf170_suppl_supplementary_materials_1-5_tables_s1-s2_figures_s40-s47.docx]

**Supplementary Material 1**

Before treatment, the cervical US examination, neck and chest CT, US-guided FNA/CNB (TA/SR group), and laboratory tests were performed for all patients. Tumor volume was calculated as V = πabc/6 (where V is the volume, a is the maximum diameter, and b and c are the other two perpendicular diameters). Neck and chest CT was performed to detect lymph node and distant metastasis. Specimens obtained through US-guided FNA or CNB were sent for cytologic or histologic evaluation, along with BRAFV600E mutation testing. Laboratory tests included thyroid function, complete blood count, and blood coagulation tests.

**Supplementary Material 2**

**Thermal ablation procedure**

TA was performed by three radiologists with over 5 years of experience in PTC ablation. Patients were positioned supine with their necks extended. The ablation site was sterilized and administered local anesthesia (0.5% lidocaine). Hydrodissection using an 18-gauge needle was performed to separate the critical structures surrounding the thyroid; a slow continuous infusion of saline (for MWA) or sterile distilled water (for RFA) was administered to maintain a safe distance of at least 0.5 cm. A mixture of lidocaine and normal saline (1:3) was administered near the thyroid capsule for topical anesthesia. A 17-gauge cooled MWA antenna with a 0.35-cm active tip (Intelligent Basic Type Microwave Tumor Ablation System, Nanjing ECO Microwave System, Nanjing, China) or a 17-gauge RFA electrode with a 0.5- or 0.7-cm active tip (Cooltip Radiofrequency Ablation System, Covidien, Dublin, American) was used for ablation. The moving-shot technique was used for ablation (1). The power for the ablation was maintained at 30W for MWA and 30-60W for RFA, based on the size of tumors. A complete ablation was confirmed when the high-echo ablation zone enveloped the entire tumor and extended at least 2 mm beyond its edge. For tumors close to the thyroid capsule (< 2 mm), ablation was performed after a thorough treatment of the adjacent capsule. Contrast-enhanced ultrasound (CEUS) using Perflubutane (SonoVue, Bracco, Milan, Italy or Sonazoid, Daiichi-Sankyo Co. Ltd., Tokyo, Japan) was performed to assess the ablation effect. If any nodular enhancement within the ablation zone or inadequate coverage of the tumor margin was detected, additional ablation was performed. After ablation, patients were monitored for approximately 30 minutes in the observation room for any potential complications (2).

**Surgical Resection Procedure**

The patient was positioned supine with neck extension. Routine sterile skin preparation was performed, and general anesthesia was administered. SR was performed by three surgeons, each with 10 years of clinical experience. Surgical strategies, including total thyroidectomy, hemithyroidectomy, or subtotal thyroidectomy, were selected in accordance with the American Thyroid Association Management Guidelines. A 5 to 8 cm transverse curved incision is made above the groin, followed by opening the midline. After identifying and protecting the recurrent laryngeal nerve (RLN) and parathyroid glands, the thyroid gland was removed. After the thyroid gland was removed, central lymph node dissection (CLND) was performed, and the incision was sutured layer by layer following complete hemostasis.

**Supplementary Material 3**

**Definition**

Local recurrence was defined as PTC confirmed by biopsy or surgery adjacent to the ablation zone. New tumor was defined as the occurrence of a newly identified malignant thyroid tumor within the thyroid gland or the original thyroid field after biopsy or surgery. LNM was defined as metastatic lymph nodes in the neck confirmed by biopsy or surgery during follow-up. Distant metastasis was identified when PTC cells were detected in organs beyond the cervical lymph nodes using CT, PET, or bone scans.

**Supplementary Material 4**

**Table S1.** Baseline patient characteristics after PSM (subgroup analysis).

|  | After PSM | | | | |  |
| --- | --- | --- | --- | --- | --- | --- |
| Variable | TA  (n=63) | SR-T2N0  (n=34) | P value | TA  (n=63) | SR-T2N1 (n=92) | P value |
| Age (years) | 40 (34,53) | 45.5 (33,58) | 0.222 | 40 (34,53) | 38 (30,51) | 0.844 |
| Sex |  |  | 0.433 |  |  | 0.456 |
| Male | 14 (22.2%) | 5 (14.7%) |  | 14 (22.2%) | 19 (20.7%) |  |
| Female | 49 (77.8%) | 29 (85.3%) |  | 49 (77.8%) | 73 (79.3%) |  |
| MD | 2.5 (2.3,2.8) | 2.5 (2.2,3) | 0.629 | 2.5 (2.3,8) | 2.5 (2.4,3) | 0.358 |

Note. —Data are medians with interquartile ranges in parentheses for continuous variables and are numbers of patients with percentages in parentheses for categorical variables. TA: thermal ablation. SR: surgical resection. MD: maximum diameter. PSM: propensity score matching.

**Supplementary Material 5**

**Table S2.** Tumor Changes in MD and Volume in the TA Group.

| Follow-up time |  | MD (cm) | P Value | Volume(mL) | P Value | Mean VRR |
| --- | --- | --- | --- | --- | --- | --- |
| Preablation |  | 2.5 (2.3,2.9) |  | 3.7 (2.8,6.4) |  |  |
| Postablation | 1month | 2.7 (2.4,3.1) | 0.283 | 4.4 (3.1,6.3) | 0.987 | -14% |
|  | 3month | 2.2 (2,2.7) | ＜0.001 | 2.4 (1.8,3.8) | ＜0.001 | 27.5% |
|  | 6month | 2 (1.4,2.3) | ＜0.001 | 1.5 (0.6,2.2) | ＜0.001 | 52.4% |
|  | 9month | 1.7 (1.3,2.1) | ＜0.001 | 1.1 (0.5,1.5) | ＜0.001 | 70% |
|  | 12month | 1.4 (1,1.9) | ＜0.001 | 0.8 (0.2,1.3) | ＜0.001 | 76.6% |
|  | 24month | 1 (0,1.6) | ＜0.001 | 0.4 (0,0.8) | ＜0.001 | 87% |
|  | 36month | 1.3 (0,1.7) | ＜0.001 | 0.6（0,1,1） | ＜0.001 | 89.7% |

Note. —Data are medians with interquartile ranges in parentheses for continuous variables and are numbers of patients with percentages in parentheses for categorical variables. MD: maximum diameter. VRR: volume reduction ratio.

**Reference**

1. Mauri G, Pacella CM, Papini E, et al. Image-Guided Thyroid Ablation: Proposal for Standardization of Terminology and Reporting Criteria. *Thyroid*. 2019;29(5):611-618. doi:10.1089/thy.2018.0604

2. Zheng L, Dou JP, Han ZY, et al. Microwave Ablation for Papillary Thyroid Microcarcinoma with and without US-detected Capsule Invasion: A Multicenter Prospective Cohort Study. *Radiology*. 2023;307(3):e220661. doi:10.1148/radiol.220661
